# Supplementary material for: Chromothripsis during telomere crisis is independent of NHEJ, and consistent with a replicative origin
Source: Genome Res. 2019 May;29(5):737–49. doi: 10.1101/gr.240705.118 (PMC6499312; doi:10.1101/gr.240705.118)
Supplement: Supplemental Material [file supp_gr.240705.118_Supplemental_file_1.zip › contigs/annotated_contigs/DB115/contig.2.DB115_length_454_mean_cov_5.88766519824.docx]

**DB115_length_454_mean_cov_5.88766519824**

```
AAAAAAAGAAAAAGAAAAAGAAAATTACCAACAAATAAAAGTCAAGGACCAGATGAATTCACAGCAGAATTTTACTAGACATTCAAGGA
 >chr3:118286983-118287201 - E=1e-116
AGAATTGGTACCAATCCTTTTGACACTATTCCAAAACGCCGAGAAAGAAGGAACCCTCCCTAAATTGTTCCATGAAGCCAGTATTGCCC

TAATACCAAAACTAGGACAGGACATAACCGAAAAAGAAA|G|TCTTGTGCCAGTTTTCAAGGGAAATGTTTCCAGCTTTTGCTTATTCA
                                        >chr3:118179407-118179644 - E=4e-128
ATATGATATTGGCCGTGAGTTTGCCATAAATAGCTCTTATTATTTCGAGATATAGTCCATCAATACCCAGTTTATTAAGAACTTTTAGC

ATGAAGCGTTGTTGGATTTTATGGAAAGCTTTTTCTGCATCTATTGAGATAATCATGTGTTTTTTGTCATTGGTTCTGTTTATGTGATG

GATTACATTTA
```
